# The Impact of Perceived Teacher Support on Reading Literacy: The Mediating Roles of Reading Self-Concept and Reading Engagement

**DOI:** 10.3390/bs16091467

**Published:** 2026-08-24

**Authors:** Xiaocheng Wang, Jiaxin Mei, Lina Jia, Liaojian Qu, Yuanying Jin

**Affiliations:** 1 Department of Education, School of Humanities, Jiangnan University, Wuxi 214122, China; 6242004017@stu.jiangnan.edu.cn (J.M.); jialina@jiangnan.edu.cn (L.J.); quliaojian@jiangnan.edu.cn (L.Q.); 2 Department of Education, School of Humanities, Sejong University, Seoul 05006, Republic of Korea

**Keywords:** perceived teacher support, reading literacy, reading self-concept, reading engagement

## Abstract

To date, limited research has systematically explored the interconnections between perceived teacher support, self-concept, engagement, and academic achievement specifically regarding the reading domain. Most previous studies have been conducted in Western cultures, making it difficult to extrapolate the findings to Eastern cultures, such as China. Therefore, this study investigated the relationship between perceived teacher support, reading self-concept, reading engagement, and reading literacy in a Chinese educational context. A structural equation modeling analysis was conducted using 12,058 Chinese students from the Programme for International Student Assessment 2018 data. The results revealed that perceived teacher support did not directly affect students’ reading literacy. However, it could indirectly affect reading literacy through the independent mediation effects of reading self-concept and reading engagement as well as their chain mediation effect. Hence, the results suggest that perceived teacher support can increase students’ sense of reading self-concept, resulting in greater reading engagement, which may lead to improved reading achievement.

## 1. Introduction

Reading literacy is an essential life skill. Attaining sufficient reading skills is considered a prerequisite for later academic performance and successful social integration (OECD, 2019a). Researchers have been trying to identify psychological and educational variables that can explain and predict variations in reading achievements (Schiefele et al., 2012). Among these variables, perceived social support has been shown to be related to students’ reading achievements. For example, students who perceive their teachers (Ma et al., 2021) or parents (X. Chen & Hu, 2021) as supportive, perform better in reading. However, it remains unclear how providing such support affects students’ progress. Existing research suggests that perceived social support may indirectly influence reading performance through affective and motivational mechanisms (Wentzel, 1998), such as self-concept (Ma et al., 2021) and engagement (Liang & Tian, 2021; Roorda et al., 2017). Nevertheless, there is insufficient empirical evidence on this subject, especially regarding Chinese students, who experience a cultural context that differs from many Western educational settings.

Given the influence of a Confucian heritage, Chinese language teachers tend to adopt a traditional teacher-directed, didactic approach to teaching reading, which over-emphasizes teaching vocabulary and the main ideas of the texts while neglecting the instruction of higher-order thinking skills and reading strategies (Law, 2011). Specifically, teachers spend considerable time delivering knowledge, for example, explaining the background knowledge, main content, and rhetorical usage of texts, along with the main goal of teaching students to achieve high scores on standardized tests. Extensive and free voluntary reading, which is important for the growth of reading competence, has been largely neglected, discouraged, and even forbidden because of intense study schedules and the lack of immediate effect on improving students’ academic achievement (Wang & Zhao, 2025). This teaching approach might efficiently transmit knowledge to students in a crowded classroom; however, it directly clashes with adolescents’ increasing need for autonomy and self-direction (Eccles & Midgley, 1989). As evidence, although Chinese 15-year-olds ranked first in reading achievement on the Programme for International Student Assessment (PISA) in 2009 (OECD, 2010), they have also exhibited motivational problems which will likely continue into adulthood (Wang & Zhao, 2025).

Even though China’s recent education reform initiatives have prompted student-centered pedagogies and encouraged teachers to improve students’ reading ability by encouraging them to read more and read independently (Ministry of Education of the People’s Republic of China, 2011), the teacher-centered lecturing and rote learning, often claimed as imprint of Confucian education culture, remains unchanged particularly at the secondary level (Zhao, 2020). The education reform failure well reflects the complex socio-political-cultural entanglements of China’s highly centralized curriculum, exam culture and high testing pressure, rigorous state control, Confucian educational tradition, large class size, teachers’ incapability and/or lack of autonomy (Deng, 2010; Zhao, 2020). The teacher-directed, authoritative teaching style also reinforces the teacher’s authority (J. J.-L. Chen, 2005). Similarly, control and governance are regarded as a part of the Chinese teacher’s responsibilities, which might explain the positive effect of teachers’ strictness (e.g., disciplining students or grading them harshly) on East Asian students’ motivation (Jiang et al., 2021). Chinese people tend to believe that “a strict teacher produces outstanding students” (严师出高徒)—an old Chinese proverb.

Based on the Confucian culture, humility and modesty are considered virtues by Chinese people. This leads Chinese students to develop a tendency to underestimate their abilities (Cai et al., 2007; S. Lau, 1996). Similarly, in Asian cultures (e.g., Chinese), as opposed to Western, self-criticism (e.g., admitting to faults) is more socially appropriate than self-enhancement (e.g., expressing positive views about oneself) (Markus & Kitayama, 1991). This is evidenced by research demonstrating that Chinese students have lower self-esteem (Cai et al., 2007), self-concept (Gu & Lau, 2023), and self-efficacy (K. Lau, 2004) compared to their Western counterparts. Similarly, self-esteem predicted outcome variables, such as life satisfaction, as worse in collectivist than individualistic cultures (Diener & Diener, 1995).

Given the above cultural and educational differences, we may question whether perceived teacher support contributes similarly to Chinese students’ reading literacy as it does to Western students’. Moreover, it would be worthwhile to examine whether this support indirectly influences reading literacy through motivational mechanisms, such as reading self-concept and reading engagement. Thus, this study attempts to investigate the mediation effects of reading self-concept and reading engagement on the association between perceived teacher support and reading literacy in a Chinese context. We believe it can help literacy researchers and educators develop a more nuanced understanding of the Chinese educational context and provide insights for teachers to design culturally adaptive interventions and tailored pedagogical strategies.

### 1.1. Perceived Teacher Support and Reading Literacy

Self-determination theory (SDT, Deci & Ryan, 1985; Ryan & Deci, 2020) dictates that to be intrinsically motivated, students’ basic psychological needs should be satisfied. These needs include autonomy (feeling a sense of volition and ownership over one’s actions), competence (feeling effective and capable, especially when facing challenges), and relatedness (feeling meaningfully connected to others). The fulfillment of these needs depends on receiving adequate support from important others, such as teachers (Ahmed et al., 2010; Diaconu-Gherasim et al., 2022; Roorda et al., 2017) and parents (X. Chen & Hu, 2021).

Perceived teacher support is considered the degree to which students believe that they can rely on their teacher’s help (OECD, 2019a). Based on the social support framework (Tardy, 1985), perceived teacher support has several dimensions, including instrumental, emotional, informational, and appraisal support. Instrumental support involves different helping behaviors, such as helping students develop skills, accomplish a difficult task, or solve a problem. Emotional support refers to the provision of trust, love, and empathy. Informational support refers to giving advice or information, while appraisal support indicates providing evaluative feedback (Tardy, 1985). Among them, emotional and instrumental supports are considered the two most important dimensions (Jiang et al., 2026).

Research has revealed that perceived teacher support, including emotional support (Jensen et al., 2019), appraisal support (Jiang et al., 2021), and instrumental support such as employing motivating instructional practices that allow students more autonomy and stimulate their curiosity to read (Law, 2011; Ma et al., 2021), facilitates students to become competent and active readers, which in turn promotes their reading achievement. It seems that the effect of perceived teacher support on students’ reading performance might be mediated by motivational and behavioral factors, such as reading self-concept (Ma et al., 2021) and reading engagement (Liang & Tian, 2021).

### 1.2. The Mediating Effect of Reading Self-Concept

Various reading-related motivation constructs drive students to engage in reading. However, reading self-concept can directly influence how students approach reading tasks and their consequent performance (Griffin et al., 2022). Self-concept pertains to individuals’ beliefs about themselves based on their interactions with the world and life experiences (Wigfield & Karpathian, 1991). Students develop a more or less domain-specific self-perception due to their experiences in different domains or subjects (Schiefele et al., 2012). Reading self-concept is “an individual’s overall self-perception as a reader, including one’s sense of competence and the role ascribed to reading as a part of one’s personal identity” (Conradi et al., 2014, p. 154). With respect to the scale of reading self-concept, it is suggested to include perceptions of competence in reading and difficulties with reading (Chapman & Tunmer, 1995).

According to the expectancy-value theory, the motivation for individuals to complete various tasks stems from their beliefs about their ability to succeed at the task and the value they attach to the task (Eccles & Wigfield, 2020; Wigfield & Eccles, 2000). Studies consistently suggest that a positive self-concept is highly associated with learning motivation, behavior, and general expectations regarding one’s performance and future (Wigfield et al., 2006). Students perceiving themselves as competent are more likely to perform better than those who do not hold such a perception, which further applies to the reading domain (De Naeghel et al., 2012; Smith et al., 2012). Therefore, establishing a positive self-concept is suggested to be the most effective way of improving reading ability (Calsyn & Kenny, 1977).

Self-concepts do not develop within students in isolation. They are highly affected by environmental factors, such as the opinions of significant others and concrete feedback (Conradi Smith & Jang, 2021). As a significant person in students’ school lives, teachers’ support plays a vital role in promoting students’ motivational beliefs such as perceived competence and task values (Diaconu-Gherasim et al., 2022; Lei et al., 2018; Patrick et al., 2007; Zhao et al., 2019). Therefore, it is plausible that reading self-concept might be a mediator between perceived teacher support and students’ reading performance. This is implied in Situated Expectancy-Value Theory (Eccles & Wigfield, 2020), which posits that students’ beliefs mediate the association between their perceptions of teacher behavior and learning outcomes. Ma et al. (2021) empirically validated this hypothesis, demonstrating that perceived teacher support positively predicts reading literacy through the enhancement of reading self-concept.

### 1.3. The Mediating Effect of Reading Engagement

Reading engagement has been defined as “interacting with texts in ways that are both strategic and motivated” (Guthrie et al., 2012, p. 602). Engaged readers are viewed as motivated, strategic, knowledgeable, and socially interactive (Guthrie & Wigfield, 2000). Reading engagement has been generally considered a multidimensional construct that consists of behavioral (students’ participation in reading activities, e.g., reading amount and diversity), affective (students’ positive/negative feelings associated with reading, e.g., reading enjoyment), and cognitive (the willingness to exert effort for better comprehension, e.g., use of cognitive strategies) subtypes (Fredricks et al., 2004; Unrau & Quirk, 2014). Recently, the social aspect (e.g., exchange of interpretations of texts with peers or teachers) has also been considered a subtype of reading engagement (Y. Lee et al., 2021). Reading engagement is highly relevant for students’ reading achievement and plays a vital role in reducing gaps between subgroups, such as socio-economically disadvantaged and advantaged groups (Kirsch et al., 2002; Wigfield et al., 2008). Guthrie and Wigfield (2000) explained that becoming engaged readers provided students with self-generated learning opportunities that were equivalent to several years of education. Reading engagement substantially compensates for low family income and poor educational background.

As noted by Sinclair et al. (2003), engagement is not an enduring attribute or a relatively stable individual characteristic of a student, but rather a state of being that is highly influenced by contextual factors such as teacher support. Research has found that perceived teacher support is significantly associated with students’ behavioral and emotional engagement in learning, such as becoming more interested in learning (Lei et al., 2018) and being motivated to endeavor for academic excellence (J. J.-L. Chen, 2005; Lei et al., 2018). However, another study found that teachers’ emotional and academic support was neither a mediator nor a direct predictor of learning engagement (Ansong et al., 2017), indicating that the relationship between perceived teacher support and learning engagement needs further research. With respect to reading, research has shown that students tend to be more behaviorally engaged in reading (e.g., spend more time reading and enrich themselves with diverse reading materials) when they perceived increased teacher support (Zhao et al., 2019). Thus, it is plausible that reading engagement might play a mediating role between perceived teacher support and students’ reading performance.

### 1.4. Reading Self-Concept and Reading Engagement

While motivation is often defined concerning an individual’s beliefs, values, and goals in a given area, engagement is a multi-dimensional construct, including behavioral, affective, and cognitive attributes associated with being deeply involved in an activity (Guthrie et al., 2012). As noted by Afflerbach and Harrison (2017), “motivation is somewhat like a reader’s potential energy: It is what you have when you are ready to read, when your reading bike is paused, as it were, at the top of a hill. Engagement is more like a reader with kinetic energy: It is manifest when the reader is zooming down the mountain bike trail of a challenging text, fully absorbed, fully engrossed, totally immersed in the activity of reading” (p. 217). Motivation is considered a mindset that can lead students to engage with reading (Afflerbach & Harrison, 2017).

As a reading-related motivation construct, self-concept is an important prerequisite for, or antecedent of, learning engagement (Patrick et al., 2007; Pekrun, 2006) as well as engagement in various areas such as reading (Unrau & Quirk, 2014; Wang, 2023), mathematics (Schnitzler et al., 2021), and science (Bae & DeBusk-Lane, 2019). Concerning reading, students who perceive themselves as capable readers tend to engage more in reading, both behaviorally (reading more) and emotionally (enjoying reading) (Locher et al., 2021). Positive reading self-concept helps foster a desire and tendency to read and enjoyment of or interest in reading (Bokhorst-Heng & Pereira, 2008). Therefore, engagement can serve as an explanatory mechanism between students’ motivational processes and academic achievements (Connell & Wellborn, 1991; De Naeghel et al., 2012).

### 1.5. Previous Studies Regarding Chinese Students

Previous studies regarding Chinese students are limited and found similar results regarding the relationships discussed above. Specifically, Chinese teachers’ support was found to be positively related to students’ motivational beliefs, such as task values and competence beliefs (Jiang et al., 2021; Zhao et al., 2019). If students perceive teachers’ concern and support, they tend to believe themselves to be competent learners and obtain positive academic emotions (Lei et al., 2018). Furthermore, J. J.-L. Chen (2005) revealed that perceived teacher support positively affected students’ academic achievement directly (*β* = 0.35, *p* < 0.001) and indirectly through the mediating effect of perceived academic engagement (*β* = 0.14, *p* < 0.01). However, using a sample of 497 middle school students in eastern China, An et al. (2023) found that perceived teacher support did not directly affect students’ academic achievement, but rather through the independent mediating effects of students’ positive emotions (*β* = 0.32, *p* < 0.001) and learning engagement (*β* = 0.075, *p* < 0.001) as well as their chain mediating effect (*β* = 0.066, *p* < 0.001).

Similar findings are also applicable to the reading domain. For example, using PISA 2018 data, Jiang et al.’s (2026) multilevel mediation model results indicated that teacher’s reading instructional practices directly (*β* = 0.06, *p* < 0.01) and indirectly affect Chinese students’ reading literacy through the mediating roles of two sub-components of reading self-concept, namely perception of competence and perception of difficulty (*βs* = 0.02, *ps* < 0.01). Others have shown that perceived teachers’ emotional and instrumental support positively affected Chinese adolescents’ reading literacy directly (*β* = 0.14, *p* < 0.01) and indirectly through the mediation role of emotional and behavioral reading engagement (*β* = 0.26, *p* < 0.001; Zhang et al., 2014). As the most relevant study to our research, Ma et al. (2021) showed that perceived teacher support not only directly, but also indirectly predicted reading literacy through the independent mediating roles of reading self-concept and emotional reading engagement, as well as their chain mediation effect. In contrast, Liang and Tian (2021) found that perceived teachers’ emotional and instrumental support only affected reading literacy via the chain mediating role of behavioral reading engagement and reading interest (*β* = 0.14, *p* < 0.01). Behavioral reading engagement by itself cannot mediate the relationship between perceived teacher support and reading literacy.

These differences might stem from the different definitions and measurements of key variables such as perceived teacher support and reading performance. For example, some studies used a combination of emotional, instrumental, and cognitive support to represent teacher support (e.g., J. J.-L. Chen, 2005), while others adopted different combinations (Liang & Tian, 2021; Zhang et al., 2014) or only instrumental support (Jiang et al., 2026). In addition, most of these studies only used one aspect, for example, the behavioral aspect (e.g., reading time, reading amount, reading diversity, Liang & Tian, 2021; Zhao et al., 2019) or the emotional aspect (e.g., reading enjoyment, Ma et al., 2021), to represent students’ reading engagement. Furthermore, both Liang and Tian (2021) and Zhao et al. (2019) considered reading interest as an individual mediator rather than as an emotional aspect of reading engagement. Although Zhang et al. (2014) used both behavioral and emotional aspects to represent reading engagement, they did not include reading self-concept in the model. Thus, the relationship among these constructs remains unclear regarding Chinese students. Notably, none of these studies have controlled for student-level variables such as students’ gender and economic, social, and cultural status (ESCS), which have been consistently shown to affect students’ reading literacy (X. Chen & Hu, 2021; Ma et al., 2021), and school-level variables such as school region, school size, class size, and student–teacher ratio, which have significant effects on students’ reading outcomes (H. Lee, 2022).

### 1.6. The Present Study

**Figure 1:**
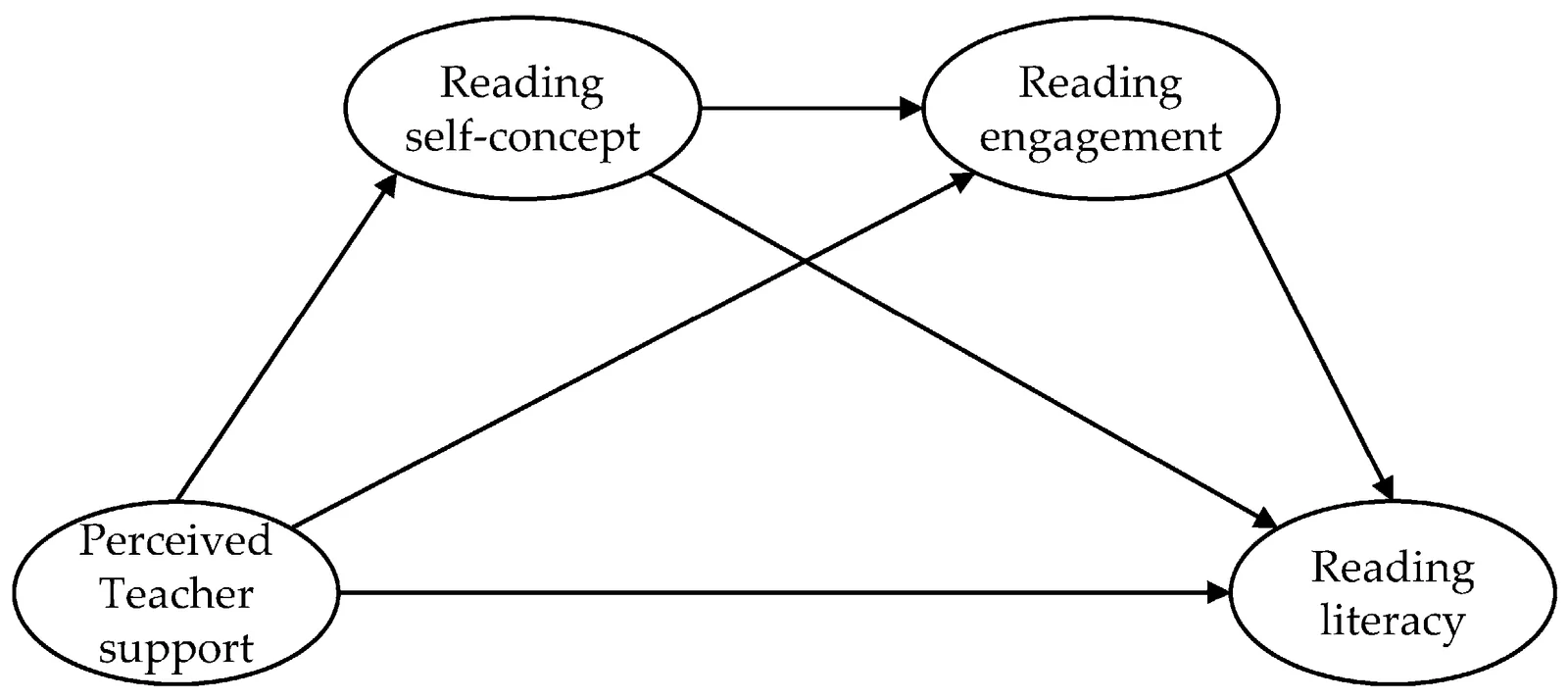
The hypothesized theoretical model.

Although there is an abundance of theoretical and empirical literature focusing on correlational and causal relationships between perceived teacher support and academic performance, and between self-concept or engagement and academic performance, little is known about the interconnection of these constructs, such as the potential explanation for the mediation mechanisms of self-concept and engagement especially concerning the reading domain. Furthermore, most previous studies have been conducted in Western contexts, making it difficult to extrapolate their findings to Eastern cultures such as China, given the aforementioned cultural and educational differences. Therefore, this study aims to investigate the relationship among perceived teacher support, reading self-concept, reading engagement, and reading literacy using a Chinese sample (Figure 1).

Based on the literature discussed above, we propose the following research hypotheses:

**Hypothesis** **1.** *Perceived teacher support positively predicts Chinese students’ reading literacy.*

**Hypothesis** **2.** *Reading self-concept mediates the relationship between perceived teacher support and reading literacy.*

**Hypothesis** **3.** *Reading engagement mediates the relationship between perceived teacher support and reading literacy.*

**Hypothesis** **4.** *Reading self-concept and reading engagement have a chain mediation effect on the relationship between perceived teacher support and reading literacy.*

## 2. Methods

A secondary analysis was conducted to test the theoretical model. Specifically, we used data from PISA, which is an international student assessment project that examines 15-year-olds’ performance in three domains—reading, mathematics, and science. PISA assesses the extent to which 15-year-old students, nearing the end of compulsory education, have acquired the essential knowledge and skills for full participation in modern societies. The assessments are developed cooperatively, agreed upon by participating countries/economies, and implemented by national organizations. In addition to tests, related factors, such as teaching and learning constructs, student background constructs, and metacognitive/non-cognitive constructs, are examined as well (OECD, 2019a). PISA 2018 is the seventh cycle since the program’s inception, mainly assessing the reading domain.

### 2.1. Participants

PISA 2018 used a two-stage stratified sampling procedure to recruit participants. A minimum of 150 schools was selected in each country/economy. Within each participating school, a predetermined number of students was randomly selected with equal probability. Four areas including Beijing, Shanghai, Jiangsu, and Zhejiang in Mainland China, participated in PISA 2018. In the first stage, schools were sampled by the probability proportionate to size sampling method, taking school characteristics, such as location, school type, and school quality, into account. Schools were excluded because they were situated in remote regions and were inaccessible, because they were very small, or because of organizational or operational factors that precluded participation. In the second stage, 15-year-old-students (ranging from 15 years and 3 months to 16 years and 2 months) were randomly sampled from each school. However, students with a functional or intellectual disability, limited proficiency in the language of assessment, currently not attending in-person classes, below 7th grade, and non-Chinese nationality were excluded from the enrollment process, resulting in an overall exclusion rate of 3.17% (OECD, 2019b). The final Chinese sample comprised 12,058 students (*M*_age_ = 15.77 years, *SD*_age_ = 0.29 years) from 361 schools. After weighing, the sample represented 992,302 15-year-olds in Mainland China.

### 2.2. Measures

In accordance with the hypothesized model, variables, including perceived teacher support, reading self-concept, reading engagement, and reading literacy, were selected from the PISA 2018 dataset (for detailed items, see Appendix A).

Perceived Teacher Support. Perceived teacher support contains four items, focusing on emotional (e.g., “The teacher shows an interest in every student’s learning”) and instrumental (e.g., “The teacher gives extra help when students need it”) support. All items were rated on a Likert-type scale, ranging from 1 (every lesson) to 4 (never or hardly ever). The Cronbach’s alpha was 0.86.

Reading Self-Concept. The reading self-concept scale includes six items, assessing the two dimensions noted by Chapman and Tunmer (1995)—perceptions of competence (beliefs about proficiency and ability; items 1, 2, and 3; e.g., “I am a good reader”) and perceptions of difficulty (beliefs that reading is problematic or hard; items 4, 5, and 6; e.g., “I have always had difficulty with reading”). Each item was rated on a Likert-type scale, ranging from 1 (strongly disagree) to 4 (strongly agree). Perceptions of difficulty items were reversely recoded. Thus, higher scores represented lower levels of perceived difficulty. Cronbach’s alphas for the two subscales were 0.79 (perceptions of competence) and 0.77 (perceptions of difficulty).

Reading Engagement. As suggested by previous research (Brozo et al., 2007; Kirsch et al., 2002), we used attitude toward reading (5 items; e.g., “Reading is one of my favourite hobbies”) and diversity of reading (5 items; e.g., “How often do you read these materials because you want to?”) to measure reading engagement. Attitude toward reading (also called reading enjoyment or reading interest; X. Chen & Hu, 2021; Ma et al., 2021; Zhao et al., 2019) corresponds to affective engagement, while diversity of reading refers to behavioral engagement. These two subtypes have been the most studied and are considered as benchmarks for evaluating the success of the other forms of engagement (Reschly & Christenson, 2012). Furthermore, studies embracing a two-component model generally include an affective and behavioral dimension (Ansong et al., 2017; Fredricks et al., 2004; Skinner et al., 2008; Unrau & Quirk, 2014). Students rated items of attitude toward reading on a Likert-type scale, ranging from 1 (strongly disagree) to 4 (strongly agree). The negatively worded items (1, 4, and 5) were reversely coded, with higher scores corresponding to more positive attitudes toward reading. Items of reading diversity were rated on a 5-point Likert scale, with higher scores representing more diverse reading. The scale’s Cronbach’s alpha was 0.73.

Reading Literacy. According to the PISA 2018 reading framework, reading literacy involves “the capacity to understand, use and reflect on written texts in order to achieve goals, develop knowledge and potential, and participate in society” (OECD, 2019a, p. 34). Based on this definition, the measurement of reading literacy contains three cognitive processes—locating information (e.g., accessing and retrieving information within a text), understanding (e.g., representing literal meaning), and evaluating and reflecting (e.g., reflecting on content and form). Both multiple-choice and open-response items were used. Reading tasks given to each student were dynamically determined based on how the student performed in previous test stages. As different items were used for different students, PISA used item response theory (IRT) techniques to calibrate item-invariant person estimates. Specifically, the IRT models produce a posterior distribution of student abilities, from which 10 plausible values (PVs) are randomly drawn as indicators of adolescents’ reading proficiency (Holland & Hoskens, 2003). Each participating student received 10 PVs, coded from PV1READ to PV10READ. Following the OECD recommendations, all 10 PVs were used to represent students’ reading literacy.

Control Variables. The control variables consist of covariates at both student and school levels. Student-level control variables included students’ gender and economic, social, and cultural status (ESCS). School region, school size, class size, and student–teacher ratio, which have significant effects on students’ reading outcomes, were chosen as school-level control variables. Student gender was measured through a single item with two categories (female = 1, male = 2). ESCS was derived from a combination of three variables—home possessions, parents’ highest occupational status, and parents’ highest level of education. Scores of these variables were used to provide a composite measure of students’ socioeconomic status in the PISA dataset. School region refers to the community in which the targeted school is located, and was measured on a Likert-type scale, with 1 = A village, hamlet or rural area (fewer than 3000 people), 2 = A small town (3000 to about 15,000 people), 3 = A town (15,000 to about 100,000 people), 4 = A city (100,000 to about 1,000,000 people), and 5 = A large city (with over 1,000,000 people). School size indicates the total number of students enrolled at a given school, while class size indicates the average size of test language classes in school. The student–teacher ratio was calculated as the proportion of total students by the total number of teachers.

### 2.3. Analysis

We conducted all statistical analyses by means of statistical software SPSS 20.0 and Amos 23.0. Before the main analysis, the missing data (approximately 3% of the entire dataset) were handled using the expectation maximization algorithm. Data were screened to check assumptions of normality. The coefficients of skewness and kurtosis were between −2.0 and +2.0.

To examine the hypothesized structural model, we conducted a structural equation modeling (SEM) analysis using the maximum likelihood estimation method. The SEM analysis involved two major sections—testing a measurement model (conducting confirmatory factor analysis [CFA] with all latent variables) and a hypothesized structural model (using an SEM analysis) (Anderson & Gerbing, 1988). Item parceling was utilized to construct the measurement model for two reasons. First, the scales of reading self-concept and reading engagement were originally developed for Western cultures and parceling can provide more stable factor solutions than item-based solutions (Little et al., 2002). Second, parceled solutions can provide better model fit because they have fewer parameters to estimate; thus, resulting in fewer chances for residuals to be correlated (MacCallum et al., 1999).

In accordance with Hu and Bentler (1999), a combination approach was used to evaluate the model fit. Specifically, two incremental close-fit indices, that is, comparative fit index (CFI) and Tucker–Lewis index (TLI), and two absolute close-fit indices, that is, root mean square error of approximation (RMSEA) and standardized root mean square residual (SRMR) were chosen. The CFI provides a measure of fit that compares the hypothesized model to a null model (i.e., no model parameters are specified) and a saturated model (i.e., all model parameters are specified). The TLI penalty increased model complexity (i.e., over parameterization) and reward increased model simplicity. The RMSEA provides a measure of fit distance from a model-implied perfect model. The SRMR evaluates the difference between the observed and predicted correlations or covariances in a model. For CFI and TLI, values higher than 0.95 and 0.90 suggest an excellent and adequate fit, respectively. For RMSEA and SRMR, values lower than 0.05 and 0.08 indicate a good and reasonable fit, respectively. Considering the large sample size (*n* = 12,058), chi-square statistics (χ^2^) was not considered a criterion to evaluate the model fit due to its sensitivity to sample size (Marsh et al., 1988).

Regarding the significance of indirect effects, we conducted bias-corrected bootstrapping. There is a 95% probability that the indirect effect is significant if the bootstrap confidence interval (CI) does not include zero (Shrout & Bolger, 2002).

## 3. Results

### 3.1. Descriptive Statistics and Correlations

Harman’s single-factor test was conducted to examine potential common method variance in the dataset. The unrotated exploratory factor analysis extracted multiple distinct factors, and the first unrotated factor accounted for 34.16% of the total variance, below the conventional 40% threshold. The results indicate that no severe common method bias exists in the current study. Table 1 shows the means, standard deviations, and correlation coefficients for all the variables. Most of the variables were significantly correlated with one another. ESCS and school region were positively correlated and were positively related to most of the variables, except class size and student–teacher ratio. School size showed a positive relationship with class size, student–teacher ratio, school region, and reading literacy; however, it was negatively related with perceived teacher support. Class size was positively associated with student–teacher ratio and negatively related with ESCS, school region, perceived teacher support, and reading self-concept. These results suggested that the larger the sizes of school and class, the less support students perceived from their teachers, which coincide with our expectations. Student–teacher ratio was negatively associated with most of the variables except for school and class sizes. Perceived teacher support, subscales of reading self-concept and reading engagement, and reading literacy were all positively correlated. Notably, perceived teacher support showed stronger correlations with students’ perceived competence and attitude toward reading. Furthermore, students’ attitude toward reading showed the strongest association with reading literacy.

### 3.2. Testing the Measurement Model

Before testing the hypothesized structural model, we examined the construct validity of the scales of perceived teacher support (one-factor model with 4 items), reading self-concept (two-factor model with 6 items), and reading engagement (two-factor model with 10 items) by conducting CFAs. The goodness-of-fit indices indicated adequate constructive validity for all the scales (Table 2). Next, we specified a full measurement model with all latent variables and performed a CFA to examine its appropriateness. Based on the rule of item-to-construct balance by adding the highest and lowest loaded items as a set (Little et al., 2002), nine parcels (i.e., three for measuring reading self-concept, three for measuring reading engagement, and three for measuring reading literacy) were constructed. According to the CFA results, the measurement model demonstrated a high level of fit to the data (Table 2). The latent factors were correlated with values ranging from 0.09 to 0.62 (*ps* < 0.001). Each item significantly loaded on its respective construct, with values ranging from 0.67 to 0.99 (*ps* < 0.001), demonstrating that the scales for measuring each construct had adequate convergent validity.

### 3.3. Testing the Hypothesized Structural Model

**Figure 2:**
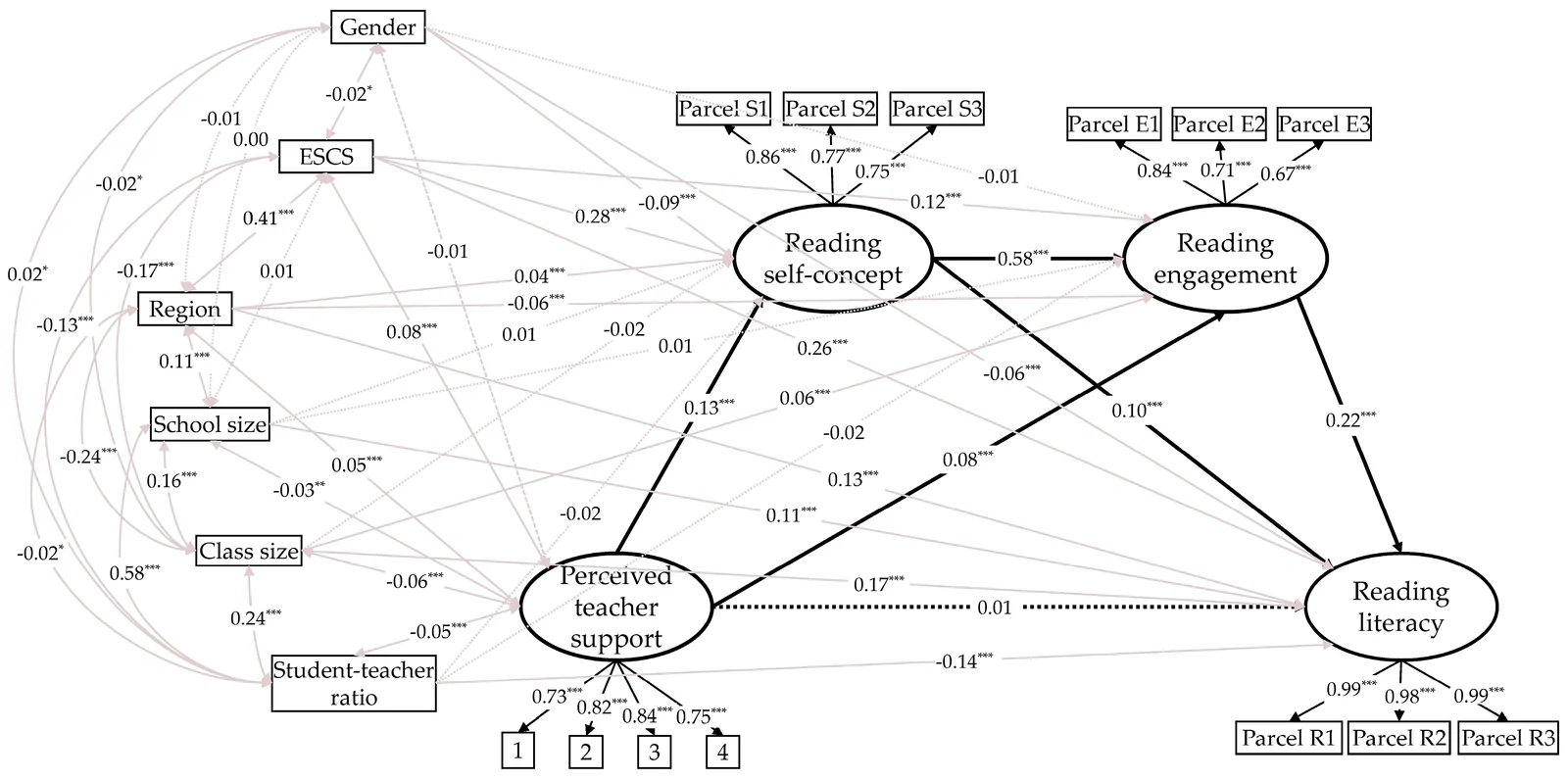
The structural model. Note. * *p* < 0.05, ** *p* < 0.01, *** *p* < 0.001. The solid arrows indicate significant influences while the dashed arrows indicate non-significant influences.

The structural model was examined and it demonstrated an excellent fit to the data, as indicated by the CFA results (Table 2). Figure 2 shows the standardized path coefficients and Table 3 summarizes the estimates of the direct, indirect, and total effects on reading literacy. Perceived teacher support did not significantly and directly affect Chinese students’ reading literacy; thus, H1 was unconfirmed. The independent mediation effects of reading self-concept and reading engagement on the relationship between perceived teacher support and reading literacy were both significant; thus, H2 and H3 were confirmed. Specifically, a 1-unit rise in perceived teacher support predicted a 1.87- and 2.60-point increase in reading literacy via the mediating roles of reading self-concept and reading engagement, respectively. The chain mediation effect of reading self-concept and reading engagement was statistically significant; therefore, H4 was confirmed. This result revealed that a 1-unit rise in perceived teacher support predicted a 2.49-point increase in reading literacy via this chain mediating pathway.

## 4. Discussion

### 4.1. The Relationship Between Perceived Teacher Support and Reading Literacy

This study examines the relationship between perceived teacher support, reading self-concept, reading engagement, and reading literacy in the Chinese context. Perceived teacher support did not directly affect reading literacy, which is consistent with An et al. (2023) and Liang and Tian’s (2021) findings, while challenging other previous results showing a direct effect of perceived teacher support on students’ reading achievements (Law, 2011; Ma et al., 2021). Three possible reasons might account for this finding. First, Chinese language classes emphasize students’ knowledge of words, idioms, grammar rules and usage, and the ability to memorize standardized interpretations of texts (Law, 2011), which are considered helpful for achieving a better score in Chinese language tests. However, according to PISA, reading literacy refers to “understanding, using, evaluating, reflecting on and engaging with texts” (OECD, 2019a, p. 28). It presents students with realistic problems and issues to solve and involves the use of higher-level reading and reasoning skills. Furthermore, reading texts used by PISA included digital, author-based, non-continuous, and multiple texts, which are not usually taught in Chinese language arts classes and are unfamiliar for Chinese students. Therefore, the support provided by the teachers, such as teacher-directed instructional practices based on prescribed texts, might not directly result in better reading literacy in PISA (Gu & Lau, 2023). Second, the scale of perceived teacher support refers to general support for students’ learning rather than focusing on reading. Moreover, as a global assessment, it does not include the unique features of Chinese teachers’ support. For example, unlike Western culture, control and governance are regarded as Chinese teachers’ responsibilities. Teacher strictness, such as harshly disciplining students or stringently grading their work (Jiang et al., 2021), might be considered a special type of teacher support by Chinese students. However, these characteristics of the Chinese educational context were not fully reflected in the present scale.

The last possible explanation might be related to the highly competitive education systems and large class sizes, due to which teachers’ support (e.g., instructional practices or feedback) is achievement-based and provided in a simple and controlling way, which can be detrimental to students’ learning outcomes (Gu & Lau, 2023). Furthermore, students tend to study in advance in order to perform better in class; thus, students who need teachers’ “extra help” (items 2 and 3) or “continued teaching” (item 4) are usually struggling or underachieving students. This explanation is supported by K. C. Lau and Lam (2017), who found similar results in PISA 2015 and proposed a possible reason that weaker students tend to receive more individual feedback from teachers. It can also find support in Qian and Lau (2022), who showed that students in schools with higher levels of directed instruction and perceived feedback tended to have lower reading performance. Hence, collectively, the expected positive effect of perceived teacher support on reading literacy may be diminished or prove to be non-significant.

### 4.2. The Mediating Role of Reading Self-Concept

The SEM results showed that although negligible in magnitude, reading self-concept effectively mediated the relationship between perceived teacher support and reading literacy. The more support students perceive from teachers (e.g., caring students’ learning and providing academic guidance to help them resolve learning difficulties), the more likely they are to believe that they are competent readers and are more likely to take the initiative to read and have more perseverance (Smith et al., 2012), which in turn leads to higher reading proficiency. The mediation effect size of reading self-concept is highly consistent with that reported in Jiang et al. (2026). This result supports previous studies demonstrating the positive effects of perceived teacher support on reading self-concept (Jensen et al., 2019; Ma et al., 2021; Zhao et al., 2019), those of reading self-concept on reading achievement (De Naeghel et al., 2012; Locher et al., 2021; Smith et al., 2012), as well as the mediating role of reading self-concept (Jiang et al., 2026).

This finding is in line with the theory of social-motivational processes, which emphasizes that students’ social world influences academic outcomes primarily through intrapersonal and psychological processes (Wentzel, 1998). Considering that humility and modesty are perceived as virtues in Chinese culture and self-enhancement is deemed less socially appropriate than self-criticism in collectivist cultures (Markus & Kitayama, 1991), we question if the effect of reading self-concept on Chinese adolescents’ reading literacy is different from that found in Western studies. However, as demonstrated by the current study, reading self-concept holds a cross-cultural positive effect (see also Jiang et al., 2026). This result particularly required teachers to protect students’ reading confidence and enhance their competence beliefs in reading by giving them sufficient praise and encouragement (Jiang et al., 2026) as well as employing motivating instructional practices (Law, 2011; Ma et al., 2021).

### 4.3. The Mediating Role of Reading Engagement

In accordance with previous research concerning general academic settings (J. J.-L. Chen, 2005; Lei et al., 2018) and the reading domain (Zhang et al., 2014), the present results support the hypothesis that perceived teacher support is indirectly correlated with reading literacy through the mediation role of reading engagement. Hence, receiving a higher level of teacher support might encourage students to read more diversely (behavioral engagement) and experience more enjoyment in reading (affective engagement), which contributes to the improvement of reading literacy. This also coincides with J. J.-L. Chen (2005), who demonstrated that the more supportive adolescents perceive their teachers to be of their education, “the more frequently they would engage in academically oriented activities” (p. 110).

However, different from previous findings, this study only found a negligible effect of perceived teacher support on reading engagement (*β* = 0.008, *p* < 0.001; see similar findings in Liang & Tian, 2021). A plausible explanation might be related to the education system, which is a didactic and hierarchical system in which teachers are the knowledge bearers and agents of knowledge transmission (Ansong et al., 2017). This learning environment does not promote active student engagement, instead, it clashes directly with adolescents’ increasing need for autonomy and self-direction (Eccles & Midgley, 1989). Furthermore, due to intense study schedules in Chinese middle schools and the competitive exanimation system, free voluntary reading has been largely neglected, discouraged, and even forbidden by teachers because of its “time-consuming” nature and lack of immediate effect on improving students’ academic achievement (Wang & Zhao, 2025). Consequently, Chinese adolescents might find it difficult to enjoy reading or develop an intrinsic interest in reading. Another possible reason might stem from the differences in class size and teacher’s workload. It is hard to expect Chinese teachers to form close relationships with students in a crowded classroom. As evidence, class size was negatively related to students’ perceived teacher support (cf. Table 1 and Figure 2).

### 4.4. The Chain Mediation Effect of Reading Self-Concept and Reading Engagement

The chain mediation effect of reading self-concept and reading engagement on the relationship between perceived teacher support and Chinese adolescents’ reading literacy was statistically significant. This result coincides with previous research showing that self-concept is an important antecedent of, or prerequisite for, student learning engagement (Patrick et al., 2007; Pekrun, 2006) as well as reading engagement (Ma et al., 2021; Unrau & Quirk, 2014; Wang, 2023). This finding supports Deci and Ryan’s (1985) self-determination theory, which hypothesized that an increase in perception of competency leads to an increase in intrinsic motivation (e.g., attitude toward reading). This view is also supported by the strong association between reading self-concept and attitude toward reading (*rs* = 0.55, 0.33, *ps* < 0.001; cf. Table 1). Overall, although perceived teacher support exerted no direct effect on reading literacy in this study, students indirectly benefited from such support by altering their motivational beliefs such as perceiving themselves as competent readers and then devoting more time to reading and enjoying reading more, which in turn positively predicted their reading literacy. In other words, the educational context does not automatically and necessarily directly affect the outcomes of reading; rather, the effects of the context ultimately rely on students’ intrinsic motivational and behavioral mechanisms. Overall, since the introduction of reading self-concept and reading engagement reduces the direct effect of perceived teacher support on reading literacy to zero, we can conclude that reading self-concept and reading engagement play a fully mediating role in the relationship between teacher support and reading literacy.

### 4.5. Limitations

There are several limitations to be noted. First, since a secondary analysis using existing database was conducted, the content and validity of the scales used in this study need further consideration. For example, the scale of perceived teacher support refers to the general support for students’ learning rather than support focused on reading (e.g., Pelletier et al., 2025). Moreover, the reading engagement scale focuses on behavioral and affective engagement in reading. Cognitive and social engagement should also be considered in future research. Furthermore, all variables are student self-reported except reading literacy. Such a methodological limitation may explain the statistically significant but substantively negligible relationship (e.g., perceived teacher support → reading engagement) and the non-significant relationship (e.g., perceived teacher support → reading literacy) found in this study. Overall, this indicates that we should build more nuanced and clearer theoretical models, clarifying the definitions, boundaries, and measurement of the key concepts in future research.

Second, the generalizability of the results is limited due to the use of a Chinese sample. Although the sample covered diverse types of schools, all participating regions are relatively developed areas with richer educational resources than other parts of China. The findings may therefore better reflect the situation of students in developed regions, and generalization to less developed areas should be made with caution. It would be worthwhile to explore the generalizability of these relationships in other regions of China, and furthermore, in different cultural contexts by using the data of other PISA participants. In addition, we did not examine whether the present relationship remains for different subgroups (e.g., proficient readers vs. developing readers). For example, reading self-concept might work differently for predicting the reading achievement of struggling readers as opposed to advanced readers (Conradi Smith & Jang, 2021). Similarly, our study focused on individual-level psychological processes rather than on school- or classroom-level effects and thus did not utilize multilevel structural equation modeling to fully account for the hierarchical structure of the data; as a result, standard errors may be underestimated, and the statistical significance of the estimates should be interpreted with this caveat in mind.

Finally, this study uses cross-sectional data. Despite a strong theoretical basis for perceived teacher support, reading self-concept, and reading engagement as antecedents for reading literacy, the direction of relationships among these variables cannot be clearly deduced. At the very least, the relationship between reading self-concept and reading engagement may be bidirectional. Thus, the directions of effects in the present model must be discussed with caution. To explore possible causal relationships, a longitudinal design is needed in future research.

### 4.6. Implications

Despite these challenges, this study contains important implications for instructional practices. First, the results highlight the importance of reading self-concept and reading engagement as mediators of the relationship between perceived teacher support and adolescents’ reading literacy. Although no direct effect of perceived teacher support on reading literacy was confirmed, the indirect pathways indicate that perceived support from teachers could enhance students’ sense of reading self-concept, resulting in greater reading engagement and ultimately leading to improved reading achievement. This reminds teachers that they should provide various types of support, such as caring about students’ feelings and needs and helping them solve reading problems, to enhance students’ sense of reading self-concept and stimulate them to engage in reading rather than solely focusing on improving reading achievements. Given the important role of students’ growth mindsets (i.e., considering one’s competence can grow and be developed), reading instruction should focus on highlighting students’ learning trajectories and growth.

Second, since the affective aspect of reading engagement (i.e., attitude toward reading) showed stronger correlations with reading self-concept and reading literacy, it is important to pay more attention to students’ positive feelings about reading. As intrinsic motivation for reading decreases during adolescence (McKenna et al., 2012; Smith et al., 2012), creating educational contexts in which students can view themselves as competent and engaged readers may be an important potential starting point. This is particularly important since perceived teacher support only showed a negligible effect on reading engagement, probably due to the instructional approach that focuses on memorization, drilling, and examination-taking coping strategies. This approach is ill-conceived if the goal of reading instruction is, at least in part, to foster students’ love for reading.

Finally, it is important to investigate the types of teacher support (e.g., emotional, informational, or instrumental) that the students benefit the most from, how the unique dimensions of teacher support contribute to reading literacy, and if the pattern holds across different cultural contexts. It is necessary to re-examine the dimensions of the perceived teacher support scale when considering that teacher’s strictness positively predicted Chinese students’ reading achievement (Jiang et al., 2021). Future research might consider developing a more validated instrument for measuring teacher support as perceived by Chinese students and its effect.

## Figures and Tables

**Table 1:** Descriptive statistics of and bivariate correlations among variables.

Variables	Mean	Range	Standard Deviation	1	2	3	4	5	6	7	8	9	10	11
1. Gender	1.52	1, 2	0.50	—										
2. ESCS	−0.36	—	1.08	−0.02 *	—									
3. School region	3.77	1–5	1.30	−0.01	0.41 **	—								
4. School size	1883.86	—	1451.47	0.00	0.01	0.11 **	—							
5. Class size	38.84	—	8.19	−0.02 *	−0.17 **	−0.24 **	0.16 **	—						
6. Student–teacher ratio	10.65	—	6.16	−0.02 *	−0.13 **	−0.02 *	0.58 **	0.24 **	—					
7. Perceived teacher support	3.39	1–4	0.69	−0.01	0.08 **	0.05 **	−0.03 **	−0.06 **	−0.05 **	—				
8. Perceived competence	2.93	1–4	0.56	−0.02 *	0.29 **	0.14 **	−0.01	−0.09 **	−0.06 **	0.16 **	—			
9. Perceived difficulty	2.84	1–4	0.63	−0.11 **	0.20 **	0.12 **	0.01	−0.05 **	−0.04 **	0.08 **	0.34 **	—		
10. Attitude toward reading	3.19	1–4	0.55	−0.17 **	0.20 **	0.05 **	0.01	0.02 *	−0.04 **	0.15 **	0.55 **	0.33 **	—	
11. Reading diversity	3.00	1–5	0.92	0.01	0.20 **	0.05 **	0.01	0.00	−0.03 **	0.11 **	0.37 **	0.16 **	0.40 **	—
Reading literacy (PV1)	561.03	—	90.34	−0.09 **	0.38 **	0.24 **	0.07 **	0.06 **	−0.09 **	0.08 **	0.21 **	0.26 **	0.34 **	0.20 **
Reading literacy (PV2)	560.27	—	90.05	−0.09 **	0.38 **	0.23 **	0.07 **	0.07 **	−0.09 **	0.06 **	0.21 **	0.27 **	0.34 **	0.21 **
Reading literacy (PV3)	560.80	—	89.83	−0.09 **	0.37 **	0.23 **	0.07 **	0.07 **	−0.09 **	0.07 **	0.21 **	0.26 **	0.34 **	0.21 **
Reading literacy (PV4)	560.27	—	89.80	−0.09 **	0.38 **	0.23 **	0.07 **	0.06 **	−0.09 **	0.07 **	0.21 **	0.26 **	0.34 **	0.22 **
Reading literacy (PV5)	560.39	—	90.02	−0.08 **	0.38 **	0.24 **	0.07 **	0.07 **	−0.09 **	0.06 **	0.22 **	0.27 **	0.34 **	0.21 **
Reading literacy (PV6)	560.84	—	89.75	−0.09 **	0.38 **	0.24 **	0.07 **	0.06 **	−0.09 **	0.07 **	0.22 **	0.27 **	0.34 **	0.21 **
Reading literacy (PV7)	560.77	—	90.24	−0.09 **	0.38 **	0.23 **	0.07 **	0.06 **	−0.09 **	0.07 **	0.22 **	0.27 **	0.34 **	0.21 **
Reading literacy (PV8)	559.40	—	89.44	−0.09 **	0.38 **	0.23 **	0.07 **	0.07 **	−0.09 **	0.07 **	0.22 **	0.26 **	0.34 **	0.22 **
Reading literacy (PV9)	560.30	—	89.73	−0.09 **	0.38 **	0.23 **	0.07 **	0.06 **	−0.09 **	0.07 **	0.21 **	0.27 **	0.35 **	0.21 **
Reading literacy (PV10)	561.08	—	90.13	−0.08 **	0.38 **	0.24 **	0.07 **	0.06 **	−0.09 **	0.07 **	0.21 **	0.26 **	0.34 **	0.21 **

Note. * *p* < 0.05, ** *p* < 0.01; ESCS = economic, social, and cultural status.

**Table 2:** Model fit indices.

Scales	χ^2^ (df)	CFI	TLI	RMSEA	SRMR
Perceived teacher support	119.160 *** (2)	0.995	0.984	0.070	0.013
Reading self-concept	163.784 *** (7)	0.993	0.985	0.043	0.015
Reading engagement	2124.653 *** (32)	0.933	0.906	0.074	0.055
**Models**					
Measurement model	856.198 *** (59)	0.994	0.992	0.033	0.021
Structural model	1360.582 *** (113)	0.993	0.990	0.030	0.017

Note. CFI = comparative fit index; TLI = Tucker–Lewis index; RMSEA = root mean square error of approximation; SRMR = standardized root mean square residual. *** *p* < 0.001.

**Table 3:** Estimates of the direct, indirect, and total effects on reading literacy.

Path	B	*β*	SE	95% CI
*Direct effect*				
Perceived teacher support → reading literacy	1.179	0.008	0.009	[−0.009, 0.025]
*Indirect effect*				
Perceived teacher support → reading self-concept → reading literacy	1.866	0.013	0.002	[0.009, 0.017]
Perceived teacher support → reading engagement → reading literacy	2.595	0.018	0.002	[0.014, 0.023]
Reading self-concept → reading engagement → reading literacy	22.277	0.129	0.008	[0.115, 0.145]
Perceived teacher support → reading self-concept → reading engagement → reading literacy	2.487	0.017	0.002	[0.014, 0.021]
*Total effect*				
Perceived teacher support → reading literacy	8.127	0.056	0.009	[0.039, 0.073]

Note. CI = confidence interval; SE = standard error.

## Data Availability

The data that support the findings of this study are openly available in the PISA 2018 database at https://www.oecd.org/pisa/data/2018database (accessed on 11 August 2024).

## Appendix A. Items of Selected Scales

**Scales**	**Items**	
Perceived teacher support	1.	The teacher shows an interest in every student’s learning.
	2.	The teacher gives extra help when students need it.
	3.	The teacher helps students with their learning.
	4.	The teacher continues teaching until the students understand.
Perceived competence	1.	I am a good reader.
	2.	I am able to understand difficult texts.
	3.	I read frequently.
Perceived difficulty	1.	I have always had difficulty with reading.
	2.	I have to read a text several times before completely understanding it.
	3.	I find it difficult to answer questions about a text.
Diversity of reading	1.	How often do you read these materials because you want to? Magazines
	2.	How often do you read these materials because you want to? Comic books *
	3.	How often do you read these materials because you want to? Fiction (novels, narratives, stories)
	4.	How often do you read these materials because you want to? Non-fiction books (informational, documentary)
	5.	How often do you read these materials because you want to? Newspapers
Attitude toward reading	1.	I read only if I have to.
	2.	Reading is one of my favourite hobbies.
	3.	I like talking about books with other people.
	4.	For me, reading is a waste of time.
	5.	I read only to get information that I need.
Note. * Item was not retained in the final model due to an extremely low factor loading.		
